# Retraction: Alterations in mGluR5 Expression and Signaling in Lewy Body Disease and in Transgenic Models of Alpha-Synucleinopathy–Implications for Excitotoxicity

**DOI:** 10.1371/journal.pone.0314138

**Published:** 2024-11-14

**Authors:** The PLOS ONE Editors

Following the publication of this article [1], concerns were raised regarding results presented in Figs 2, 6, 7, and S4B. Specifically,

- There appear to be similarities between lanes within numerous panels presented in:
  ○ Fig 2G
  ○ Figs 6B, 6H
  ○ Figs 7G 7H
- There appear to be vertical irregularities suggestive of splice lines within panels presented in Figs 6B and 6G
- There appear to be repetitive regions within multiple panels presented in Fig S4B

Regarding the similarities between and within the western blot panels, the corresponding author stated that the quality of the western blot panels presented in the article is not optimal and that close examination of these panels shows subtle differences between the lanes and bands that appear similar. Furthermore, the corresponding author commented that the vertical irregularities may be due to rearrangement of lanes to support illustration and regrets that splice lines were not clearly marked to indicate that these lanes represent individual, but not consecutive, lanes originating from the same blot. The corresponding author did not provide high resolution image data underlying the published western blot results. PLOS remains concerned that the areas in the Fig 2, 6, and 7 panels listed above appear more similar than would be expected from independent results, and in the absence of uncropped high resolution image data, these concerns cannot be resolved.

Regarding the repetitive regions in the Fig S4B panels, the corresponding author suggested that these repetitive patterns might have occurred whilst they tried to improve the images for legibility, as the original images contained some annotations that were cut out. The corresponding author provided underlying images containing labels and markings in areas where repetitive patterns were identified in Fig S4B. PLOS therefore considers the concerns with this figure resolved.

In light of the remainder of the above concerns that call into question the reliability and integrity of the published western blot results, the *PLOS ONE* Editors retract this article.

EM did not agree with the retraction. PD responded but expressed neither agreement nor disagreement with the editorial decision. DLP, ER, KU, VP, NML, DA, AC, BS, CP, and MHE either did not respond directly or could not be reached.
